# Text-Guided Inpainting for Aesthetic Prediction in Orthofacial Surgery: A Patient-Centered AI Approach

**DOI:** 10.3390/medicina61122075

**Published:** 2025-11-21

**Authors:** Vincenzo Abbate, Emanuele Carraturo, Domenico Benfenati, Sara Tramontano, Luigi Angelo Vaira, Stefania Troise, Gabriele Canzi, Giorgio Novelli, Daniela Pacella, Cristiano Russo, Cristian Tommasino, Riccardo Nocini, Antonio M. Rinaldi, Giovanni Dell’Aversana Orabona

**Affiliations:** 1 Head and Neck Section, Maxillofacial Surgery Unit, Department of Neurosciences, Reproductive and Odontostomatological Sciences, University of Napoli Federico II, 80131 Naples, Italy; vincenzo.abbate@unina.it (V.A.); emanuele.c2971995@gmail.com (E.C.); sara.tramontano94@gmail.com (S.T.); giovanni.dellaversanaorabona@unina.it (G.D.O.); 2 Department of Electrical Engineering and Information Technology (DIETI), University of Napoli Federico II, 80138 Napoli, Italy; domenico.benfenati@unina.it (D.B.); cristiano.russo@unina.it (C.R.); cristian.tommasino@unina.it (C.T.); antoniomaria.rinaldi@unina.it (A.M.R.); 3 Maxillofacial Surgery Operative Unit, Department of Medicine, Surgery and Pharmacy, University of Sassari, 07100 Sassari, Italy; luigi.vaira@gmail.com; 4 School of Biomedical Science, Biomedical Sciences Department, University of Sassari, 07100 Sassari, Italy; 5 Maxillo-Facial Surgery Unit, ASST Grande Ospedale Metropolitano Niguarda, 20162 Milan, Italy; gabriele.canzi@ospedaleniguarda.it; 6 Operative Unit of Maxillo-Facial Surgery, Fondazione IRCCS San Gerardo dei Tintori, 20900 Monza, Italy; giorgio.novelli@unimib.it; 7 Department of Public Health, University of Napoli Federico II, 80131 Naples, Italy; daniela.pacella@unina.it; 8 Unit of Otolaryngology, Head and Neck Department, University of Verona, 37129 Verona, Italy; riccardo.nocini@gmail.com

**Keywords:** artificial intelligence, generative AI, orthofacial surgery, virtual surgical planning, CAD-CAM

## Abstract

*Background and Objectives:* Artificial intelligence (AI) is increasingly impacting medicine by improving healthcare delivery and simplifying diagnostic and therapeutic processes. Text-guided inpainting is a promising tool in orthofacial surgery for generating ideal, patient-specific facial profiles. *Materials and Methods:* A total of 89 patients with dentofacial deformities (DFDs) were evaluated. The DALL-E2 platform was used to generate profilometric transformations based on textual prompts. The resulting images were assessed by three groups: patients, expert surgeons, and the general population. *Results:* A total of 94% of surgeons, 85% of the general population, and 79% of patients rated the AI-modified profiles as more aesthetically pleasing than the originals. The prompt inspired by runway models had the highest agreement across groups. *Conclusions:* Generative AI and text-guided inpainting show potential for enhancing aesthetic planning in orthofacial surgery, offering personalized treatment paths and aiding virtual surgical planning.

## 1. Introduction

Artificial intelligence is playing an increasingly defined role in medicine, ensuring an improvement in healthcare provision as well as a simplification of diagnostic and therapeutic procedures [1]. Among the AI applications, great value is being placed on synthography. Synthography refers to the use of synthetic data generation techniques, primarily driven by AI and machine learning, to create realistic, artificial representations of complex systems, including images and other forms of data [2]. Recent advancements in deep generative models, such as Generative Adversarial Networks (GANs) and Diffusion Models, have enabled the creation of highly realistic synthetic images that closely resemble real-world medical data. These techniques are increasingly applied in medical imaging, from enhancing training datasets for AI models to generating patient-specific visualizations for surgical planning [3,4]. Synthography is a novel AI-driven technique that involves the generation of synthetic data, primarily images, using advanced neural network models. These models learn to create highly realistic representations by analyzing large amounts of real-world data and capturing underlying patterns. GANs operate through a two-network system: a generator that creates synthetic images and a discriminator that evaluates their realism—iteratively improving the quality of generated outputs. Diffusion Models, on the other hand, progressively refine noisy data through a series of denoising steps, enabling the generation of highly detailed and diverse images. In the context of medical applications, synthography allows for the creation of synthetic yet anatomically accurate images, which can be used to train AI models, augment datasets, and facilitate patient-specific visualizations. Synthography has great potential in precision medicine, particularly in surgical planning, where personalized and data-driven approaches are essential for optimizing clinical outcomes to have a patient-centered aesthetic assessment. Specifically, the ability to generate images from textual descriptions through synthography could have significant clinical implications in orthofacial surgery [5,6]. In such cases, the therapeutic goal is the restoration of facial aesthetics and occlusion in patients affected by dentofacial deformities (DFDs) [7]. In recent years, CAD-CAM technologies have revolutionized the way this pathology is treated. Through virtual surgical planning (VSP), the surgeon has the opportunity to virtually plan the movements of the bone bases and visualize the effects of these movements on the facial soft tissues, thus predicting the aesthetic changes in the facial profiles [8,9]. Although very advanced, this technology still has some limitations related to the lack of an ideal and personalized profilometric reference on which to base the VSP [10]. Cephalometry and the surgeon’s own aesthetic preference have been the only references for VSP for years. Today, it is a shared opinion that such strategies are unable to meet specific patient aesthetic demands [11]

Artificial intelligence (AI) is increasingly influencing facial surgery across pre-operative planning, morphometric analysis, and outcomes prediction. In orthognathic care, AI enables rapid, reproducible landmarking, assists automated soft-tissue simulation, and supports objective aesthetic assessment from 2D photographs and 3D scans, complementing virtual surgical planning (VSP) workflows [4,5,6,7,9,10,11].

In clinical orthodontics and maxillofacial surgery, profiles are often described as concave, convex, or straight, with sagittal tendencies such as retrognathic and prognathic patterns (including mixed or asymmetric variants), which provide a shared frame for aesthetic appraisal and treatment goals.

Recent work shows that AI can reproduce or surpass manual cephalometric landmarking and profile analysis, improving consistency across operators; deep-learning systems have also been used for automated surgical tracings and outcome prediction after orthognathic surgery, accelerating workflows while reducing variability [7,10,11]. Based on these premises, synthography could meet the need for increasingly tailored treatments aimed at ensuring a functional and aesthetic result more readily accepted by the patient, free from operator-dependent influences [2]. This work proposes and feasibility-tests a synthography-based, patient-specific profiling protocol, reports usability and acceptability, and defines a priori efficacy endpoints for future validation.

## 2. Materials and Methods

### 2.1. Cohort Selection

This is a retrospective chart review and survey carried out between 1 May 2024 and 1 June 2024 at Maxillofacial Surgery Unit in Federico II University Hospital. A cohort of 89 patients who suffered from DFDs awaiting surgery (as yet unoperated) were enrolled. Inclusion criteria were as follows:

- Age ≥ 18 years.
- Clinical and radiological diagnosis of DFDs.
- Patients whose profilometric photographic documentation was available.
- Patient who provided availability for the investigation and signed informed consent.

Exclusion criteria

- Age < 18 years
- Prior orthognathic surgery or facial implants altering profile aesthetics.
- Syndromic craniofacial disorders or major craniofacial trauma; chronic bone disease.
- Inadequate or non-standardized photographs (see protocol) or missing key clinical data.
- Active dermatological/soft-tissue conditions profoundly altering facial contours.
- Refusal of image use for research.

Patients who had undergone previous surgeries on facial hard and soft tissues, or who had chronic bone disease and/or suffered from craniofacial malformations or pathologies deforming facial morphology, were excluded from the study. All clinical investigations and procedures were conducted according to the principles expressed in the Declaration of Helsinki. Ethical approval for the study was granted by the Ethical Committee of the Federico II University Hospital (Approval No. 189/2023).

### 2.2. Study Design

**Figure 1:**
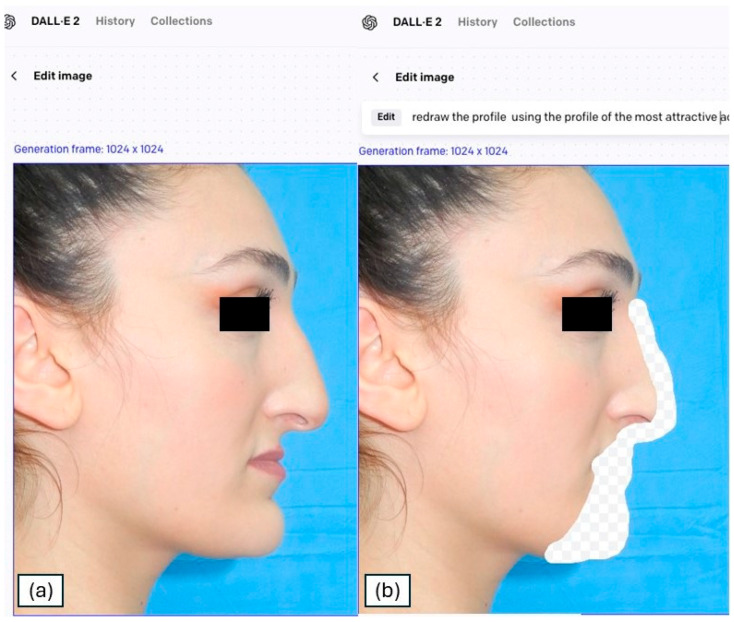
Original profile photo of the patient (**a**) and area selected for transformation on the patient profile (**b**).

To generate profilometric photographic transformations, the OpenAI online platform DALL-E2 was used in this study. OpenAI’s DALL-E2 [12] represents a cutting-edge text-to-image model that employs sophisticated deep-learning methodologies to generate high-fidelity, photorealistic imagery from natural language descriptions. Building upon the foundations laid by its predecessors, DALL-E2 has been shown to possess a substantially enhanced capacity to interpret nuanced prompts, thus facilitating the production of more accurate and detailed visual representations. In particular, DALL-E2 has demonstrated enhanced capabilities in rendering human features, such as hands and faces, addressing limitations observed in earlier versions. This enhancement is particularly advantageous for applications such as inpainting, where the model fills in missing or modified regions of an image, facilitating the creation of patient-specific ideal facial profiles in medical and aesthetic contexts. Integrating DALL-E2 into existing workflows enables professionals to achieve precise and personalized visualizations, thereby enhancing the planning and outcome of facial reconstructive procedures. For each patient, a side-view photo was taken in Natural Head Position as described by Solow and Tallgren (1971) and Lundström et al. (1995), using a FULLHD (24.2 MegaPixels) camera (Sony Alpha ZV-E10) [13,14]. The photos were then imported into the DALL-E2 platform. Through the “edit” function, the portion of the profile subject to feasible surgical changes was selected using the brush tool (Figure 1a,b).

**Figure 2:**
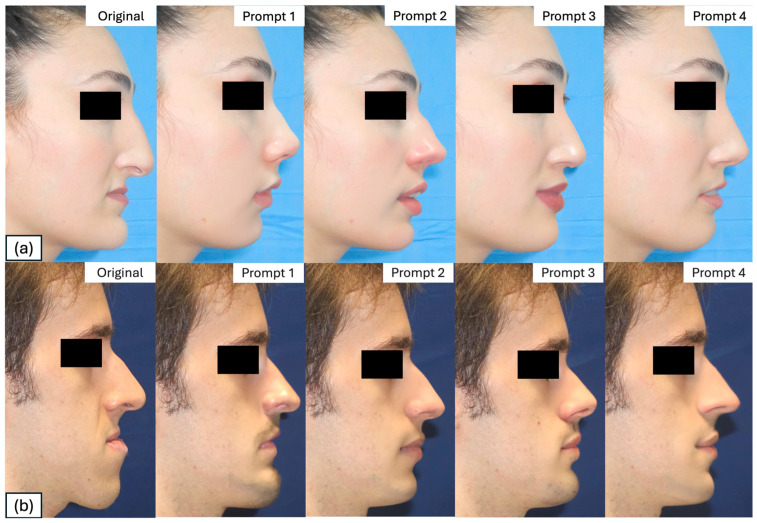
Series of transformations based on the 4 prompts for the female (**a**) and male (**b**) patient.

For each prompt used to generate transformations, the DALL-E2 generates four different proposals. The single transformation that was judged more positively on the basis of surgical feasibility and naturalness was selected from a single operator, being an expert surgeon. Then, four AI-generated photographic transformations based on the prompts defined below were collected from each patient. For all transformations, the same computer was used (Dell Precision 5540—i7-9750H). Each input prompt (Table 1) allowed the creation of a patient-specific transformed profile (Figure 2a,b).

### 2.3. Google Form Survey

**Figure 3:**
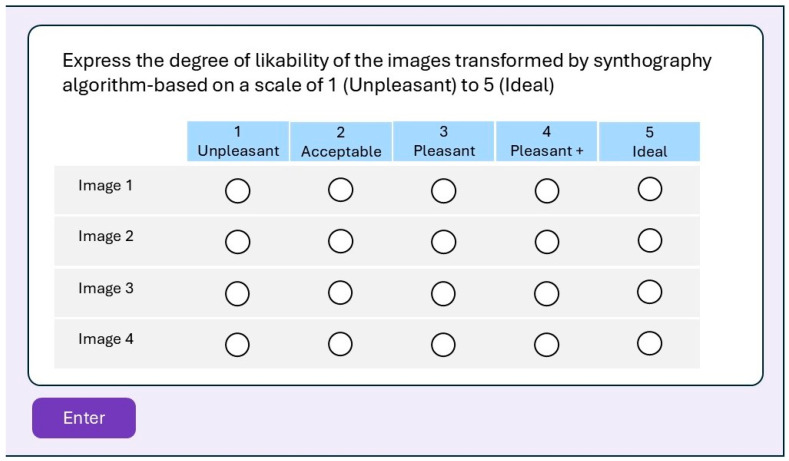
Process of obtaining modified images by means of a generative model guided by the textual prompt: from the image, the operational zone is defined for the model, which, using the textual prompt, applies the changes in the chosen zone, producing a modified image.

Through a system based on the “Google Form” model, an anonymized form containing five different profiles was created for each patient. Specifically, the first image displayed corresponds to the original profile of the patient, while the subsequent four display transformations obtained from DALL-E2 based on the described prompts but not identifiable (blinded). Additionally, each form included a dichotomous (yes/no) question about the overall perception of the attractiveness of the transformations compared to the original profile, and a “5-point Likert scale” to express the degree of preference for each transformation (Figure 3).

The survey was forwarded to the following evaluation groups.

- Patient-specific Group (PsG): The group composed of the 89 DFD patients on the waiting list to undergo surgery selected by the study. Each patient received the form containing the transformations of their own profile.
- Expert Group (EG): The group composed of eight selected specialists in maxillofacial surgery from different institutions worldwide, experts in orthognathic surgery who agreed to participate in the study. Each expert received the form containing the transformations related to the profile of the 89 DFD patients selected. The group was randomly selected in terms of gender, ethnicity, and religious belief. All group members had to have more than 10 years of surgical experience.
- General population Group (GpG): The group composed of six randomly selected individuals from the clinical database of the maxillofacial surgery unit who agreed to participate in the study. Each person received the form containing the transformations related to the profile of the 89 DFD patients selected. These individuals, patients treated or to be treated for other illnesses in our institute, were randomly selected from the point of view of education, religious beliefs, gender, and ethnicity, to obtain as heterogeneous a group as possible that would resemble a general population of common individuals.

**Figure 4:**
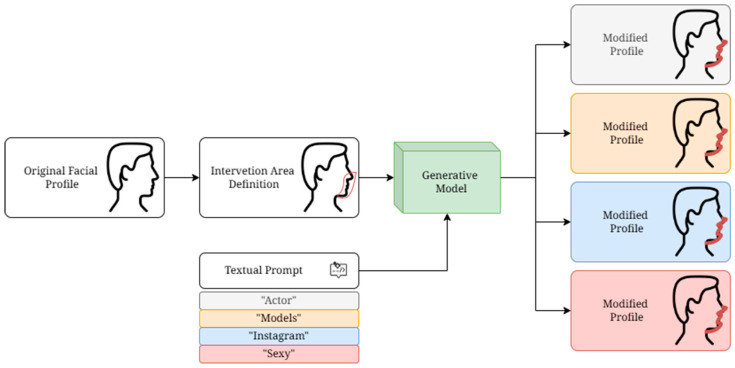
Example of the “5-point Likert scale” designed to express the degree of preference for each transformation.

Figure 4 shows the entire proposed process for obtaining the modified facial profile.

### 2.4. Statistical Analysis

Data are presented as absolute frequency and percentage for categorical variables, while they are presented as mean and standard deviation for continuous variables. Agreement between the patient’s evaluation and the surgeon’s and non-clinician’s evaluation is measured using absolute count, overall raw agreement, and Cohen’s kappa coefficient. Cohen’s kappa can be interpreted as follows: <0.20, poor agreement; 0.21–0.40, fair agreement; 0.41–0.60, moderate agreement; 0.61–0.80, good agreement; >0.81, very good agreement [10]. Paired differences were considered as the difference between the patient’s evaluation and the means of the evaluations provided for the same image on the same patients by either surgeons or non-clinicians. To evaluate the significance of the paired differences, the Wilcoxon matched-pairs signed-rank test was used. The significance level for all analyses was set to α = 0.05. All analyses were performed using statistical software R, version 4.4.0.

### 2.5. Definitions

Dentofacial deformity (DFD) in this study denotes skeletal malocclusion patterns (Class II/III, vertical dysplasia and/or transverse discrepancy), including cases with clinical asymmetry, that warranted orthodontic/orthognathic assessment. Unless otherwise specified, DFD cases were non-syndromic. Syndromic phenotypes with major craniofacial growth disturbance (e.g., Parry–Romberg syndrome, Crouzon syndrome) were excluded a priori to avoid confounding in AI-based aesthetic assessments.

#### Photography Protocol

All photographs were acquired by the same operator in the same room using the same camera/lens and fixed lighting against a neutral background. Subjects were positioned in Natural Head Position (NHP) with inter-pupillary plane horizontal, lips at rest and teeth in habitual occlusion; camera-to-subject distance and height were standardized via tripod and floor marks; and exposure and white balance were fixed for each session [13,14].

Social-media subset and aesthetic label. Instagram images were identified from public profiles meeting inclusion criteria (no filters/obvious edits; orientation comparable to clinic protocol). The label “sexy profile” was applied when ≥2 independent raters (OMFS/orthodontics background) agreed that the profile reflected contemporary attractiveness cues (e.g., harmonious naso-labial and mento-labial angles, balanced projection, cervico-mental definition) on a 5-point Likert scale (threshold ≥4/5); discordant ratings were adjudicated by a third rater.

## 3. Results

### 3.1. Cohort Results

A total of 89 patients responded to the survey by filling out the Microsoft Form, making the Patient-specific Group (PsG). The cohort consisted of 45 male and 44 female patients, diagnosed with dentofacial deformities (DFDs) and who underwent surgical procedures: specifically, 24 patients had a dentoskeletal class II, 63 patients a dentoskeletal class III, and 2 had a severe transverse maxillary deficiency. The average age of the cohort was 28.4 ± 7.2 years. Eight maxillofacial surgeons not belonging to the operating unit where the data were collected were enrolled to respond to the survey on the same Microsoft Form regarding the transformations of the 89 patients. Six external patients making up the General population Group (GpG) not suffering from DFDs and randomly selected from clinical databases collected in the operating unit to which the study belongs were also enrolled to give their opinion about the transformations through the same Microsoft Form.

### 3.2. Survey Results

**Figure 5:**
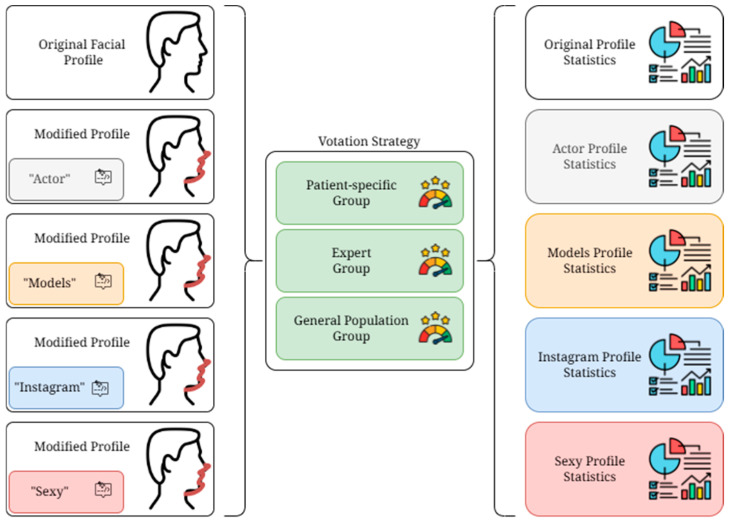
Scheme for the evaluation strategy: votes from patients, experts, and the general population are combined to abstract satisfaction metrics of the modified faces.

The analysis of the responses regarding the question “Given the previous images, do you think the following profiles modified by artificial intelligence techniques are more pleasing than the original?” showed that 21% of the Patient-specific Group (PsG) responded negatively after viewing the proposed AI transformations of his/her face. On the other hand, only 5.2% of the Expert Group (EG) and 11.5% of the General population Group (GpG) gave a negative response to the same question. Moreover, 79% of patients responded positively to the question, as well as 85% of the external population and 94% of the surgeons’ population (Table 2). Figure 5 shows the evaluation process about the survey.

Overall raw agreement and Cohen’s kappa coefficient analyzed for the PsG/EG pairs showed Poor concordance (<0.20) of responses in 75% of cases and Fair concordance (0.21–0.40) in 25% of cases.

The same analysis for the PsG/GpG pairs showed a Poor concordance (<0.20) in 33.4% of cases and a Fair concordance (0.21–0.40) in 66.7% (Table 3).

The sum of the averages of the Likert scores calculated on the individual images analyzed in the three groups showed that the most liked transformation corresponds to Prompt 1 (Profile Most Liked Actors/Actresses) followed by Prompt 4 (Profile Most Liked Models/Models), Prompt 2 (Profile Influencers with Most Followers), and lastly Prompt 3 (Profile of the Sexiest Women and Men) (Table 4).

Looking at the paired differences between the different cohorts, it is shown that the biggest difference (maximum disagreement) between the patient-surgeon cohort appears from Prompt 3 (Sexiest transformation) and the lesser (maximum agreement) for Prompt 4 (Runway Model transformation). Between the PsG/GpG cohorts, it appears that the biggest difference is seen for Prompt 3 and the smallest for Prompt 4. Between the EG/GpG cohort the biggest difference is seen for Prompt 2 and the smallest for Prompt 1. Looking at the total overall, the transformation that gained the biggest disagreement between the three verification groups (PsG/GpG/EG) appears to be the “sexiest” (Prompt 3) (Table 5).

## 4. Discussion

The management of dentofacial deformities (DFDs) has long been a topic of interest in the literature [1,2,3]. Initially, the primary therapeutic goal was to restore occlusion through jaw repositioning, without considering the aesthetic effects on facial soft tissues [4]. However, in the 1990s, the concept of orthofacial surgery was introduced by Arnett, shifting the focus from a purely functional approach to one that prioritized both aesthetic and functional outcomes [15,16]. In orthofacial surgery, the concepts of beauty and harmony have become increasingly crucial, as the main aim is to correct dentoskeletal malformations while maximizing the overall facial attractiveness of the patient [17]. Nowadays, the introduction of digital workflows and virtual planning in orthognathic surgery has generated the first major revolution for the treatment of DFDs. VSP has gradually replaced traditional analog planning by offering the surgeon the ability to digitally visualize the effect of facial bone displacement on soft tissue and thus on the patient’s profile [5,18,19]. The digital workflow begins with the digital acquisition of the patient’s morphovolumetric data through 3D optical scans and CT of the patient. The data imported into virtual planning-specific software generate a virtual twin of the patient on which surgical displacements of the bony bases and the resulting soft tissue effects are simulated. The VSP through 3D-printed patient-specific implants (PSIs) is finally transferred to the operating room on the patient to faithfully reproduce the preoperative planning [6]. Computer-assisted planning based on 3D imaging simplifies cephalometric analysis, splint production, and surgical simulation. As reported by Park et al., the integration of virtual surgical planning, made possible by 3D imaging and 3D printing, provides surgeons with a better visualization of anatomical structures and has led to substantial improvements in treatment outcomes [7]. Although all of these applications have enabled significant advances in surgical planning, defining an ideal standard of beauty tailored to the individual remains a challenge [20,21,22,23]. Beauty is a fascinating and universal concept that has captivated humanity for millennia. It is a subject rich in nuances and complexities, as its definition varies greatly from person to person, culture to culture, and across historical eras. Even today, virtual surgical planning (VSP) is guided by the specific preferences of the operating surgeon and cephalometric analysis, which remains the only objective parameter used to inform bone displacements. However, as noted, cephalometry is based on population averages and thus reveals significant variations between ethnicity and geography [24]. These differences are crucial for an accurate diagnosis and treatment planning in orthodontics and orthognathic surgery. For example, Black men exhibit different sellanasion-subspinale and sella-nasion-supramentale angles compared to Caucasian and Hispanic men, indicating skeletal differences. In addition, Japanese subjects have a more retruded chin position and steeper mandibular plane compared to Caucasians, demonstrating geographic-specific differences in cephalometric measurements. Furthermore, North Indians have more convex faces and protrusive lips compared to Caucasians, highlighting significant deviations in cephalometric standards [25,26,27,28]. Based on these considerations, artificial intelligence can offer help through synthography. The term “synthography” is new in the medical and surgical fields and lacks a clear and precise definition. However, it can be understood as a concept that encompasses techniques such as digital rendering, applications of artificial intelligence in visual creation, and the manipulation of images beyond traditional photography to produce artwork or visual results not achievable through conventional photographic methods alone. Reinhuber et al. [2] were the first to describe this concept in the informatics field, defining synthography as the method of synthetically generating digital images using machine learning. It is distinguished from other graphic creation and editing methods using text-to-image artificial intelligence models for synthetic media generation, commonly achieved through textual descriptions of “prompt engineering” as a human input to create or modify a desired image [9].

A recent bibliometric analysis of the 100 most-cited articles on artificial intelligence in orthognathic surgery and orthodontics, conducted by Ka Fai Wong et al. [29], revealed that, from 2017 to 2022, there was a notable increase in the adoption of AI, presumably linked to the growing use of new AI technologies in clinical practice. However, none of the applications described involve the use of synthography or AI techniques used to identify ideal facial profiles [29,30]. Synthography, the synthesis of medical images using AI and deep learning, has transformative applications in medicine. Here are the key uses:

- Brain MRI Enhancements: Synthography tools like SynthSR improve brain MRI scans by converting low-resolution images into high-resolution T1-weighted scans. This enables more accurate morphometric analysis and helps to study neurological conditions such as Alzheimer’s disease and brain tumors [31].
- MR-to-CT Image Translation: Synthography creates CT images from MRI scans, improving diagnostic capabilities without additional radiation exposure. Techniques such as multi-resolution networks outperform traditional methods in synthesizing wholebody CT scans [32].
- Radiotherapy Planning: Synthetic CT (sCT) images are vital for radiation therapy planning when MRI is preferred for soft-tissue contrast. Approaches such as SynthRAD2023 challenge advance adaptive radiotherapy techniques [33].
- Synthetic Pathology for AI Training: Generative models create synthetic chest X-rays to balance datasets and enhance AI’s diagnostic performance. This reduces data imbalance and privacy concerns [34].
- Tumor Synthesis for AI Model Training: Synthetic tumors generated for various organs improve AI models for tumor detection and segmentation in various imaging protocols [35].

To the best of our knowledge, there is no study in the literature on the use of synthography in facial surgery. This research therefore aims to investigate, evaluate, and validate the possible use of AI and specifically synthography in orthofacial surgery. From the analysis of the study results, the most interesting element concerns the opinions provided by the members of the three evaluation groups (PsG; EG; GpG) regarding the question “Given the previous images, do you think the following profiles modified by artificial intelligence techniques are more pleasing than the original?”.

In this regard, profile transformations were considered an improvement on the original image in 94% of EG, 85% of GpG, and 79% in PsG evaluations. Although, overall, these percentages suggest the efficacy of the tool in generating more appreciable profiles, the highest percentage of disapproval was surprisingly noted in the group of DFD patients (PsG). The same degree of disagreement was evidenced when comparing the agreeableness of the prompts considered. In particular, Prompt 3 (sexiest profile) had an overall score on the 5-point Likert scale of 2.37 for the PsG, 3.25 for the EG, and 2.89 for the GpG. Although it is the prompt that received the lowest scores overall (5.76 overall), it is also the prompt that generated the most disagreement among reviewers (1.77 overall paired differences). This “most negative” finding, predominantly in the PsG, could reflect the disperceptive nature of the DFD patients.

As shown in the literature and highlighted in [36], up to 13% of DFD patients have disorders related to self-image dissipation requiring targeted behavioral treatment, so-called Body Dysmorphic Disorder (BDD). BDD is a mental health condition characterized by an obsessive focus on one or more perceived flaws in physical appearance, which are either minor or not observable to others. Individuals with BDD can spend hours a day thinking about their perceived defects, leading to significant distress and impairments in daily functioning. In DFD patients, this disorder is more highlighted. This finding may be behind such discrepant registrations as those witnessed in this study. Likewise, a high propensity in the EG towards a sexy profile (Prompt 3) has been recorded. This finding could be explained by the fact that surgeons are accustomed to aesthetic demands more directed towards this profile in the general treatment population, resulting in a biased mindset conditioned in the choice of a given sexy profile. In the group of DFD patients (PsG), the most highly rated transformation was the one related to Prompt 4 (Model) with a score of 2.79 (1.27). The same transformation was rated 3.26 (0.71) by the EG and 2.88 (0.65) by the GpG. In general, Prompt 4 was recognized as the most highly rated prompt in the three groups (9.27). The present study aims to evaluate and validate whether AI can also be a valid tool from the point of view of VSP in orthofacial surgery. Specifically, synthography in the sense of AI-based image editing capabilities could support the prediction of changes in patient profiles planned with one of the virtual planning softwares for orthognathic surgery, which still show a limitation in post-operative soft tissue prediction. Nevertheless, synthography provides the candidate with aesthetic targets but cannot replace soft-tissue prediction models or clinical examination; AI images are presented with uncertainty ranges and a VSP-verified feasible envelope.

The present study is not exempt from limitations. First, participants were asked just once, at the beginning, to indicate whether the AI-generated images were aesthetically better than the original profile. This did not allow us to assess whether all or just a few of the proposed images were actually better than the actual patient’s profile. Additionally, the form asked for a quantitative evaluation only of the AI-generated images but did not ask the responders to quantitatively assess the patient’s aesthetic without modifications; this did not allow us to compare the scores and thus analyze whether the modified images constituted an improvement in comparison with the actual patient’s profile. Beyond global judgements of attractiveness, specific angular relationships plausibly influence the ratings derived in our pipeline. Prior work implicates the naso-labial and mento-labial angles in perceived middle–lower facial balance, while the cervico-mental angle informs submental definition and jawline aesthetics. In the present study, we evaluated facial profiles from images but did not quantify these angles; future iterations will extract them automatically and test associations with AI-based aesthetic scores and clinical outcomes.

This study is also limited by the retrospective design and reliance on 2D photographs, which cannot fully capture 3D soft-tissue behavior. The AI was trained/validated on a single-center dataset, which may constrain generalizability. Although photography was standardized, residual variance in pose/lighting cannot be fully excluded for social-media images. The exclusion of syndromic phenotypes improves homogeneity but limits applicability to atypical craniofacial types. We did not yet analyze angular metrics (naso-labial, mento-labial, cervico-mental); these will be incorporated prospectively.

## 5. Conclusions

The results of this study suggest how artificial intelligence, and specifically synthography, may prove useful in generating profilometric transformations in DFD patients that the patients themselves evaluate positively. Studies with a larger sample size are needed to confirm that the model profile (Prompt 4) can be considered an ideal reference profile in DFD patients. In this feasibility study, a text-guided inpainting protocol generated patient-specific profile variants that were generally preferred across patients, surgeons, and the public. While these findings indicate acceptability and potential usability, clinical efficacy—defined as improved, surgeon-achievable planning accuracy and patient-reported outcomes—was not tested here and remains for prospective validation alongside virtual surgical planning (VSP). The aim of future studies should be to exploit the potential of synthography to help the surgeon in preoperative virtual planning in orthognathic surgery and, moreover, in surgeries dedicated to profilometric improvement.

## Figures and Tables

**Table 1:** Prompt used to generate the profilometric transformations through the DALL-E3 platform.

Prompt	Female Patient	Male Patients	Transformation Profile Keyword
1	“redraw the profile using the sideview of the most attractive actresses as a reference”	“redraw the profile using the sideview of the most attractive actors as a reference”	Actors/resses
2	“redraw the profile using the sideview of the women with the most followers on Instagram as a reference”	“redraw the profile using the sideview of the men with the most followers on Instagram as a reference”	Instagram
3	“redraw the profile using the sideview of the most of the sexiest women as a reference”	“redraw the profile using the sideview of the most of the sexiest men as a reference”	Sexiest
4	“redraw the profile using the sideview of the most beautiful runway models as a reference”	“redraw the profile using the sideview of the most beautiful runway models as a reference”	Models

**Table 2:** Total agreement and disagreement to the question “Given the previous images, do you think the following profiles modified by artificial intelligence techniques are more pleasing than the original?” for the PsG, EG, and GpG.

Expert Group (EG)									
**Answer**	**Surgeon 1**	**Surgeon 2**	**Surgeon 3**	**Surgeon 4**	**Surgeon 5**	**Surgeon 6**	**Surgeon 7**	**Surgeon 8**	**Total**
NO	5 (5.6%)	16 (18%)	4 (4.5%)	1 (1.1%)	1 (1.1%)	9 (10%)	6 (6.7%)	1 (1.1%)	5.2%
YES	84 (94%)	73 (82%)	85 (96%)	88 (99%)	88 (99%)	80 (90%)	83 (93%)	88 (99%)	94%
General population Group (GpG):									
**Answer**	**Extern 1**	**Extern 2**	**Extern 3**	**Extern 4**	**Extern 5**	**Extern 6**			
NO	19 (21%)	16 (18%)	11 (12%)	14 (16%)	13 (15%)	7 (7.9%)			11.5%
YES	70 (79%)	73 (82%)	78 (88%)	75 (84%)	76 (85%)	82 (92%)			85%
Patient-specific Group (PsG)									
**Answer**	**Average**								
NO	19 (21%)								
YES	70 (79%)								

**Table 3:** Overall raw agreement on the answers “yes” and “no” to the question “Given the previous images, do you think the following profiles modified by artificial intelligence techniques are more pleasing than the original?” among the patients, surgeons, and external population.

			Patient-Specific Group (PsG)			
			NO	YES	Raw Agreement	Cohen k
Expert Group (EG):	Surgeon 1	NO	3	2	79.7%	0.18
		YES	16	68		
	Surgeon 2	NO	6	10	74.2%	0.18
		YES	13	60		
	Surgeon 3	NO	3	1	80.8%	0.2
		YES	16	69		
	Surgeon 4	NO	0	1	77.5%	<0.01
		YES	19	69		
	Surgeon 5	NO	1	0	79.8%	0.08
		YES	18	70		
	Surgeon 6	NO	4	5	77.5%	0.17
		YES	15	65		
	Surgeon 7	NO	4	2	80.9%	0.24
		YES	15	68		
	Surgeon 8	NO	1	0	79.8%	0.08
		YES	18	70		
General population Group (GpG):	Extern 1	NO	6	10	74.2%	0.18
		YES	13	60		
	Extern 2	NO	6	5	79.7%	0.29
		YES	13	65		
	Extern 3	NO	6	8	76.3%	0.22
		YES	13	62		
	Extern 4	NO	6	7	77.4%	0.24
		YES	13	63		
	Extern 5	NO	4	3	79.7%	0.22
		YES	15	67		
	Extern 6	NO	0	0	78.7%	<0.01
		YES	19	70		

**Table 4:** Descriptive Likert scale mean scores for each image and group.

Transformation Profile	PsG *N* = 89	EG *N* = 89	GpG *N* = 89	Total
Actor/Actress	2.79 (1.13)	3.36 (0.72)	3.04 (0.69)	9.19
Instagram	2.57 (1.14)	3.31 (0.69)	2.92 (0.66)	8.8
Sexiest	2.37 (1.00)	3.25 (0.69)	2.89 (0.63)	5.76
Model	2.79 (1.27)	3.26 (0.71)	2.88 (0.65)	9.27

**Table 5:** Paired differences between the ratings given by the patient to him-/herself and the average of the ratings given to that patient by surgeons or outsiders.

	Paired PsG/EG Differences		Paired PsG/GpG Differences		EG/GpG Differences		Total
Transformation Profile	Mean (SD)	*p*-Value	Mean (SD)	*p*-Value	Mean (SD)	*p*-Value	
Actor/Actress	+0.57 (1.24)	**<0.001**	+0.26 (1.28)	0.084	+0.31 (0.56)	**<0.001**	**+**1.14 (3.08)
Instagram	+0.73 (1.16)	**<0.001**	+0.35 (1.08)	**0.005**	+0.39 (0.55)	**<0.001**	**+**1.47 (2.79)
Sexiest	+0.88 (1.02)	**<0.001**	+0.52 (1.09)	**<0.001**	+0.37 (0.58)	**<0.001**	**+**1.77 (2.69)
Model	+0.47 (1.33)	**0.003**	+0.09 (1.40)	0.531	+0.38 (0.56)	**<0.001**	+0.94 (3.29)

## Data Availability

The original contributions presented in the study are included in the article, all the new data are presented in the manuscript.

## Abbreviations

The following abbreviations are used in this manuscript:

GANs	Generative Adversarial Networks
DFD	dentofacial deformities
VSP	virtual surgical planning
EG	Expert Group
GpG	General population Group
